# Effect of a patient-centred deprescribing procedure in older multimorbid patients in Swiss primary care - A cluster-randomised clinical trial

**DOI:** 10.1186/s12877-020-01870-8

**Published:** 2020-11-16

**Authors:** Stefan Zechmann, Oliver Senn, Fabio Valeri, Stefan Essig, Christoph Merlo, Thomas Rosemann, Stefan Neuner-Jehle

**Affiliations:** 1 Institute of Primary Care, University of Zurich, University Hospital Zurich, Pestalozzistrasse 24, 8091 Zurich, Switzerland; 2 Institute of Primary and Community Care, Lucerne, Switzerland

**Keywords:** Primary care, Older patient, Deprescribing, Public health, Randomised controlled trial, Effectiveness, Polypharmacy

## Abstract

**Background:**

Management of patients with polypharmacy is challenging, and evidence for beneficial effects of deprescribing interventions is mixed. This study aimed to investigate whether a patient-centred deprescribing intervention of PCPs results in a reduction of polypharmacy, without increasing the number of adverse disease events and reducing the quality of life, among their older multimorbid patients.

**Methods:**

This is a cluster-randomised clinical study among 46 primary care physicians (PCPs) with a 12 months follow-up. We randomised PCPs into an intervention and a control group. They recruited 128 and 206 patients if ≥60 years and taking ≥five drugs for ≥6 months. The intervention consisted of a 2-h training of PCPs, encouraging the use of a validated deprescribing-algorithm including shared-decision-making, in comparison to usual care. The primary outcome was the mean difference in the number of drugs per patient (dpp) between baseline and after 12 months. Additional outcomes focused on patient safety and quality of life (QoL) measures.

**Results:**

Three hundred thirty-four patients, mean [SD] age of 76.2 [8.5] years participated. The mean difference in the number of dpp between baseline and after 12 months was 0.379 in the intervention group (8.02 and 7.64; *p* = 0.059) and 0.374 in the control group (8.05 and 7.68; *p* = 0.065). The between-group comparison showed no significant difference at all time points, except for immediately after the intervention (*p* = 0.002). There were no significant differences concerning patient safety nor QoL measures.

**Conclusion:**

Our straight-forward and patient-centred deprescribing procedure is effective immediately after the intervention, but not after 6 and 12 months. Further research needs to determine the optimal interval of repeated deprescribing interventions for a sustainable effect on polypharmacy at mid- and long-term. Integrating SDM in the deprescribing process is a key factor for success.

**Trial registration:**

Current Controlled Trials, prospectively registered ISRCTN16560559 Date assigned 31/10/2014.

The Prevention of Polypharmacy in Primary Care Patients Trial (4P-RCT).

**Supplementary Information:**

The online version contains supplementary material available at 10.1186/s12877-020-01870-8.

## Background

Management of multimorbid patients, the majority of whom are treated by primary care physicians (PCP), is challenging [1–4]. A major issue in this predominantly old population is polypharmacy, commonly defined as the intake of five or more drugs per capita, which entails the risk of adverse drug reactions [5–8]. This subsequently leads to an increase in morbidity [9, 10], hospital admissions [11–13], health-related costs and ultimately, the number of deaths [14–16].

Several clinical deprescribing tools and strategies for different healthcare providers (pharmacists, physicians, nurses) [17–25], mostly for the inpatient setting and using computer-assisted decision aids, have been tested [26]. The communication about patients’ needs and preferences in the process of deprescribing is challenging and a patient-centred approach, taking into account these topics, is of utmost importance to enable safe and effective deprescribing [27–31]. One of the first landmark trials using this approach by Garfinkel and Mangin in 2010, introduced the Good Palliative-Geriatric Practice (GPGP) algorithm [32]. Studies with PCP-lead interventions in the primary care setting, including shared-decision-making (SDM) and long-term, follow up are still scarce.

According to previous studies of deprescribing efficiency, the impact of these interventions on clinically relevant endpoints, e.g. falls, use of health care facilities, morbidity or mortality, is often small or even nonexistent and varies [18, 19, 33, 34]. Furthermore, complex deprescribing interventions are challenging to implement in the primary care setting, e.g. due to time restrictions or structural shortcomings [17, 35, 36]. Thus, there is a need for effective and feasible approaches to reduce inappropriate polypharmacy in primary care, with an emphasis on acceptance and implementation into routine care [28, 37]. Therefore we designed a foreshorten, paper-based intervention to tackle two of the major barriers in the primary care setting especially common in Switzerland, time and lack of uniform digital solutions in practices [38, 39]. This study aimed to investigate whether a patient-centred deprescribing intervention of GPs results in a reduction of polypharmacy, without increasing the number of adverse disease events and reducing the quality of life, among their older multimorbid patients.

## Methods

### Design

We conducted a two-armed, cluster-randomised, clinical trial in Swiss primary care practices from January 1, 2015, to June 1, 2017.

### Participants

We invited German-speaking, officially registered PCPs working in ambulatory primary care practices from northern Switzerland stepwise by canton (region) by letter to participate. We stratified enrolled PCPs as clusters by practice to avoid contamination. Approximately one-half of the clusters were randomised to the intervention (*n* = 22) and one-half to control group (*n* = 24). After randomisation PCPs received the allocated training session (see below), before identifying eligible patients by reviewing their daily patient list. We asked PCPs to recruit a maximum of 15 patients. They received an expense allowance of 100 CHF per recruited patient.

**Fig. 1:**
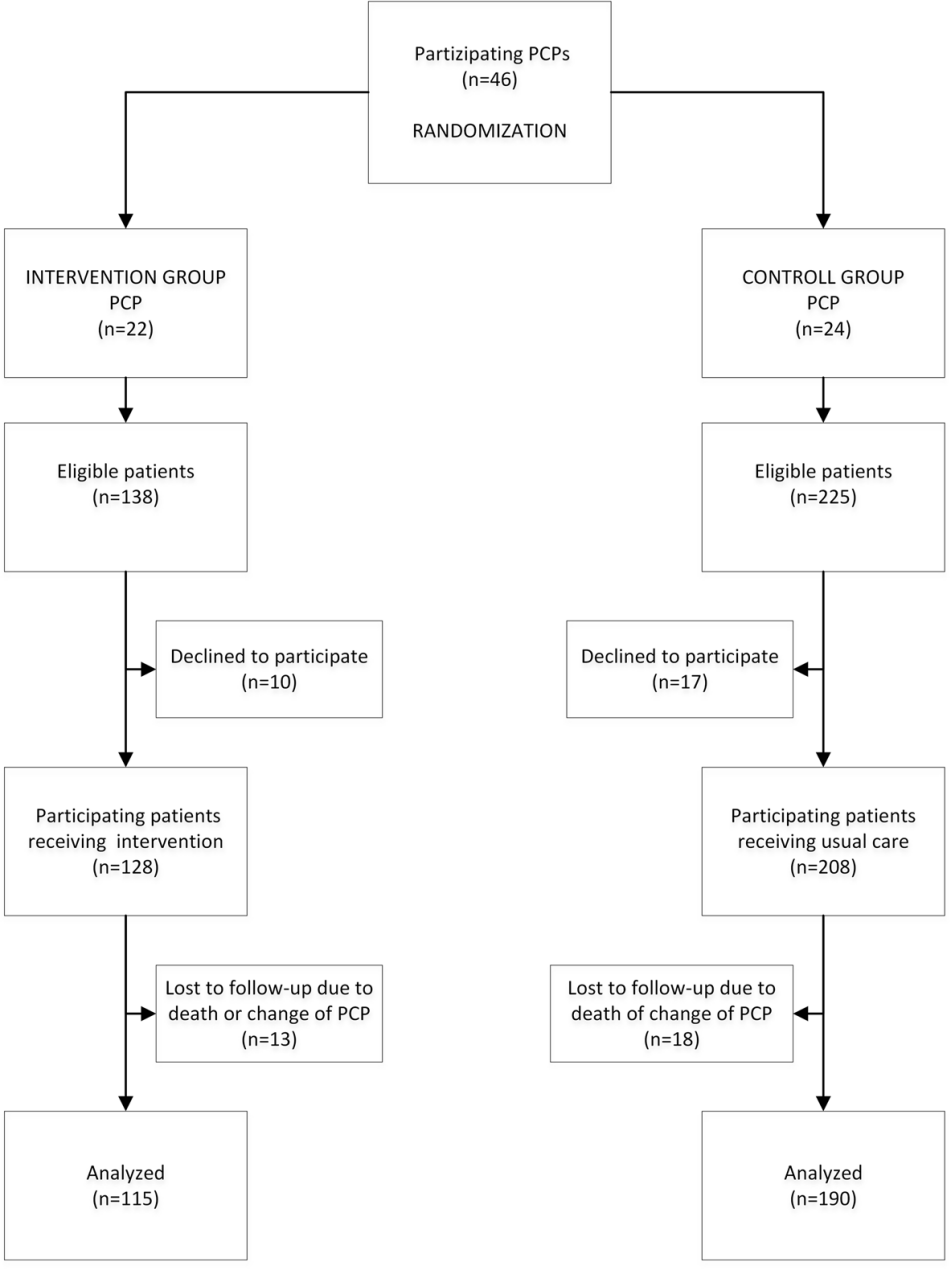
Study flowchart. Randomisation on PCP level, patient recruitment and follow-up during the study

PCPs continuously recruited patients independent of race or ethnicity whenever they fulfilled the following criteria: a) cared for by one of the participating PCPs b) ≥60 years of age c) ≥ 5 chronic drugs for ≥ 6 months d) capable of judgement. Enrolled patients were blinded to the study arm insofar as blinding was guaranteed by randomisation on PCP level. See the study flowchart to visualise the patient selection process (Fig. 1).

### Intervention description

#### Intervention on PCP level (training session)

**Fig. 2:**
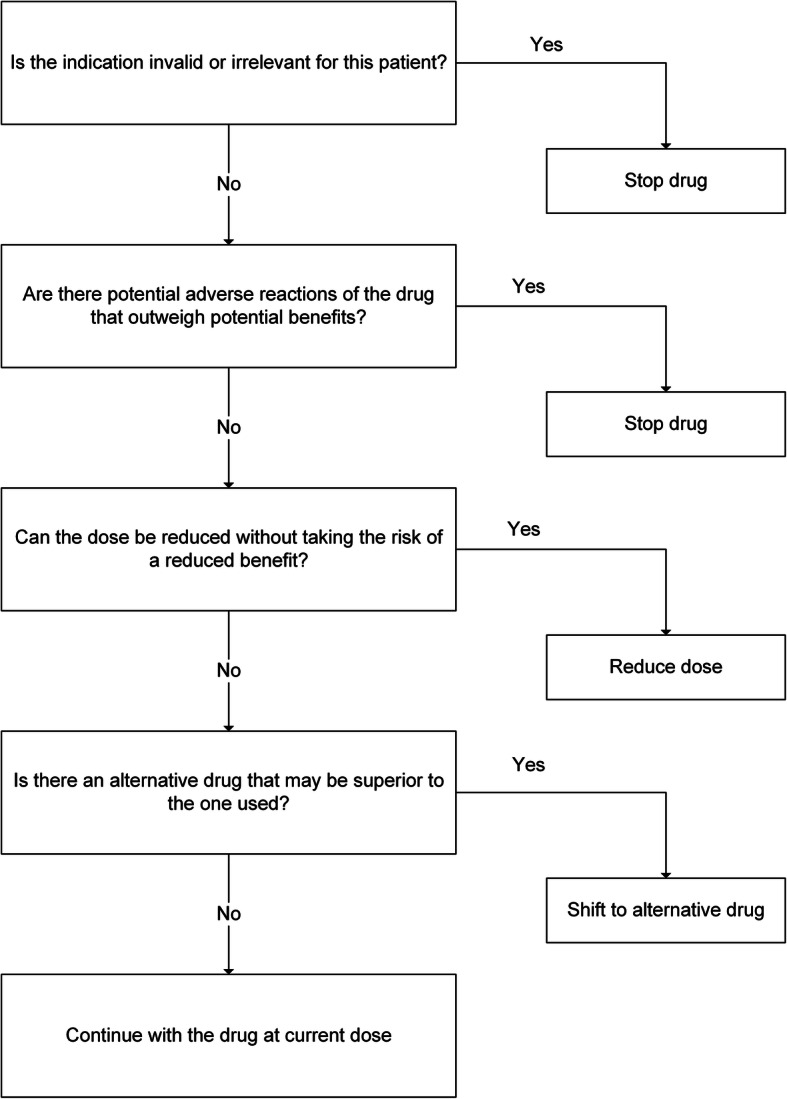
Deprescribing-algorithm. Deprescribing-algorithm used by the PCP in the intervention group

*In the intervention group*, PCPs received a lecture (length: 2 h) on polypharmacy and trained the use of our deprescribing-algorithm and to discuss its results with their patients (SDM). This intervention could take place immediately after obtaining informed consent or in a separate consultation. We adapted the Good Palliative-Geriatric Practice (GPGP) algorithm, which is a deprescribing tool developed for and tested in a geriatric setting [32]. Adaption meant simplifying from six to four key questions to answer. See Fig. 2 for details. We previously pilot-tested this adapted algorithm to assess its feasibility and practicability in the primary care setting [40].

*In the control group*, PCPs received a general lecture (length: 2 h) on multimorbidity and instructions for collecting usual care data. For blinding purposes, we informed PCPs that the study purpose was to investigate best practices for physician-patient communication.

#### Intervention on patient-level (during the first encounter)

After obtaining informed consent from the patient, a practice-nurse, or the PCP created a list of the patient’s current medication. Then, the PCP defined the four main diagnoses and a list of the four most important complaints in consensus with the patient, to facilitate prioritisation of treatment goals. PCPs in the intervention group then decided for every single drug listed if it was appropriate for the patient, according to the algorithm (Fig. 2).

Based on these questions, the PCP made a recommendation for each drug. In an SDM procedure that followed the PCPs recommendation, the PCP and patient then decided together what changes to implement, always with the option to restart if symptoms should increase or if the disease should deteriorate. PCPs used a checklist, including many individual steps to guide the first consultation (see Additional file 1 for details). The SDM procedure was guaranteed by the stepwise character of the algorithm, including the task of SDM itself.

### Time points and outcomes

Time points are defined as follows:

Pre-Intervention T0 (before the first consultation, previously also named baseline), Post-Intervention T1 (at the end of the first consultation), after 6-Months T2, and after 12-Months T3.

## Primary outcome

Mean difference in the number of drugs per person (DPP) between Pre-Intervention and after 12 months.

## Secondary outcomes

Mean difference in the number of dpp between Pre-Intervention and Post-Intervention

2.1 Mean difference in the number of dpp between Pre-Intervention and after 6 months
2.2 Number of drugs with PCP’s recommendation to change and what kind of change (either stopping, dose change or alternative drug)
2.3 Frequency of discrepant decisions between PCP and patient about the change of a drug, and therapeutic groups of drugs patients were not willing to change
2.4 Number of dpp taken without the PCPs knowledge, at Pre-Intervention
2.5 Symptom scores, rates of hospitalisation, death and unexpected clinical events
2.6 Quality of life rating by the patients at Pre-Intervention, after 6 and 12 months
2.7 Time consumption due to the intervention, by the practice nurse and by PCP

For further details, including interim analysis and stopping guidelines, see study protocol [41].

### Measures

PCPs and patients collected data on pre-coded paper documents (Additional file 1) at the four-time points. PCPs stored the codes identifying the individual patient in their practice. Data transfer to the electronic database was carried out and double-checked by two research associates.

We categorised diagnoses according to an adapted version of a classification system by van den Bussche et al. [42] For coding of complaints we used ICPC-2 coding [43] and for drugs the ATC code [44]. We compared diagnosis and complaints as perceived by PCPs and patients based on ratings. (For details and results see [45]). Patients reported QoL, as well as the symptom score of the worst complaint on paper format all three consultations. We used three different tools to document QoL:

- five-point Likert-scale ranging from − 2 to + 2, with − 2 the worst possible, + 2, the best possible,
- a visual analogue scale (VAS) ranging from 0 to 100 to document their current health status, with 0 the worst possible, 100 the best possible, and
- the “functional” EQ-5D-3L set of questions concerning topics of mobility/activity, self-catering, general activities, pain/physical complaints and fear/depression [46]. Patients reported symptoms on a scale ranging from 0 (no symptoms) to 10 (worst symptoms possible). We asked PCPs to report all hospitalisations and deaths as well as all other clinical events they considered essential for our study on a pre-coded paper form, on which more detailed information on the events` character, the context and the consequences were required, as well as the PCPs probability estimate of the causal relationship between the previous deprescribing procedure and the event. In case of drop- out, patients̀ data gathered so far were computed.

Additionally, to those characteristics above, we collected the following patient and PCP characteristics. For patients: Age [years], sex (PCP classified), number of drugs and living situation. For PCPs: Age [years], sex (investigator classified), working experience [years], type of practice [single or group] and affiliation to physician network [yes or no]. We used the web-based data management program SecuTrial® (version 5.0.1, 2016) [47]. We applied CONSORT guidelines for reporting of all data and visualisation of study flow [48].

### Statistical analysis

We randomised in blocks, making sure PCP’s from the same practice were in the same group to prevent contamination. Freely available software named “randomization.com” was used [49].

We performed power calculations for the study’s primary outcome, with a power of 80%, a two-sided alpha-error of 2.5%, a cluster effect of 2% [50], a standard error of 2.8 and an assumed effect size of 0.8 dpp (based on our pilot) [40] and an assumed drop-out rate of 10%.

For the descriptive analysis of PCPs’ and patients’ characteristics, we used frequencies and percentages for categorical variables, and for continuous variables means and standard deviation (SD) or median and interquartile range (IQR). To compare groups with categorical variables, we used the Chi-square test or Fisher’s exact test. For comparison of groups and times with continuous variables, we used Welch’s t-test or Mann-Whitney-U test. We provided *p*-values and 95% confidence intervals (95%-CI). We used the statistical program R® Version 3.5 37.

As a sensitivity analysis, we calculated the deprescribing rate, defined as the proportion of the number of drugs stopped at a given time point in relation to the number of drugs at baseline, for each time point after Pre-Intervention. We compared the rates between groups using Chi-square test or Fisher’s exact test. The distribution of drugs which were recommended to change by the PCP and those with a patient’s agreement to the recommendation was calculated.

## Results

Mean age of the 334 included patients (128 intervention group/ 206 control group) was 76.2 (SD 8.5) years, 136 (45.5%) females, taking 8.05 (SD 2.5) dpp, with no significant differences between groups. Drop-out rates were 9.3% (31 of all patients), 6.0% (20 of all patients) due to death and 3.3% (11 of all patients) due to the change of attending PCP. Mean age of 46 participating PCPs was 49.4 (SD 9.3), 30 (65%) males, working for 13.6 (SD 9.7) years in private practice. (For further details see Additional file 2 and (25)) The main therapeutic drug classes were cardiovascular (30% of all drugs), neurologic-analgetic (15.6%) and anticoagulant (8.7%) drugs at baseline. Rates of drugs stopped were 41.7% for cardiovascular, 14.8% for neurologic-analgetic and 8.3% for anticoagulant drugs. For further details on drugs, see Additional file 3.

**Fig. 3:**
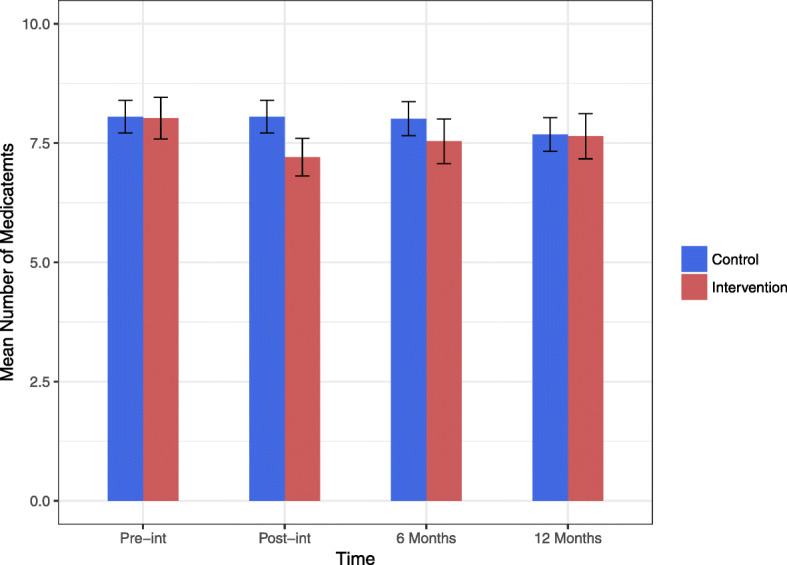
Mean number of drugs. Mean number of drugs in both groups during the study with CI (95%). X-axis = Mean number of drugs, y-axis = Time. Time points: Pre-int = Pre-intervention, Post-int = Post-Intervention

The mean difference in the number of dpp between Pre-Intervention and after 12 months (primary outcome) was 0.379 in the intervention group (8.02 at Pre-Intervention and 7.64 after 12 months; *p* = 0.059) and 0.374 in the control group (8.05 at Pre-Intervention and 7.68 after 12 months; *p* = 0.065). For the time points Post-Intervention and after 6 months, there was a significant reduction in dpp in the intervention group (p = < 0.001 and 0.002). In the control group, there was no significant change in the number of dpp over all time points. In the between-group comparison, there was no significant difference at all time points, except for Post-Intervention (*p* = 0.002) (Fig. 3). For further details, see Table 1.

**Table 1:** Mean number of drugs in both groups

	Intervention group mean dpp (95% CI)	Control group mean dpp (95% CI)	Difference between groups mean dpp (95% CI) *P*-value	
**Pre-Intervention** (T0)	8.02 (7.59–8.46)	8.05 (7.71–8.40)	−0.03 (− 0.59–0.53)	0.916
**Post-Intervention** (T1)	7.20 (6.81–7.60)^a^		−0.85 (−1.38–0.32)	0.002
**6 months** (T2)	7.54 (7.07–8.37) ^a^	8.01 (7.56–8.37)^b^	− 0.47 (− 1.07–0.12)	0.118
**12 months** (T3)	7.64 (7.17–8.12) ^a^	7.68 (7.32–8.00) ^b^	−0.04 (− 0.63–0.56)	0.906

The mean number of drugs per patient (DPP) at all four-time points and between-group differences. T0 = First consultation, before intervention, T1 = First consultation, immediately after the intervention, T2 = Second consultation 6 months later, T3 = Third consultation 12 months later. CI = 95% confidence interval, ^a)^ *p*-values for the time point comparison within the intervention group: T1-T0, T2-T0 and T3-T0: < 0.001, 0.002 and 0.059. ^b^) p-value for the time point comparison within the control group: T2-T0 and T3-T0: 0.960 and 0.065

The deprescribing rates at T2 and T3 were between 23.9 and 29.0% in the intervention group and between 14.0 and 21.5% in the control group (*p* < 0.001 for difference) (Additional file 4).

Intervention group PCPs recommended changes of drugs in 209 (20.5%) of all 1019 drugs at baseline. In 170 (81.3%) of these 209 recommendations, patients implemented a change. One hundred five drugs were stopped, and 85 (81%) of them remained stopped after 12 months.

**Fig. 4:**
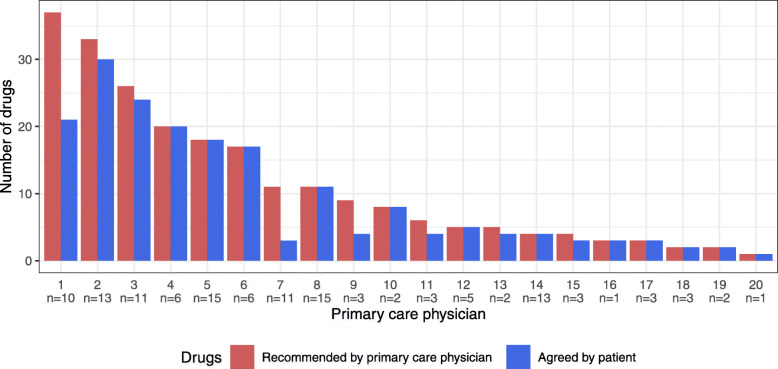
Recommended drug changes and agreement. Number of drug changes recommend per primary care physician during the first consultation (T0). This recommendation Red bar shows the number of drugs recommend for change by the primary care physician and the blue bar shows the number of drugs on which the patient and the primary care physician agreed to proceed with the recommended change

On PCP level, the agreement of patients to the recommended drug changes was 86% (weighted mean per centre 0.858, SD 0.231) (Fig. 4).

**Fig. 5:**
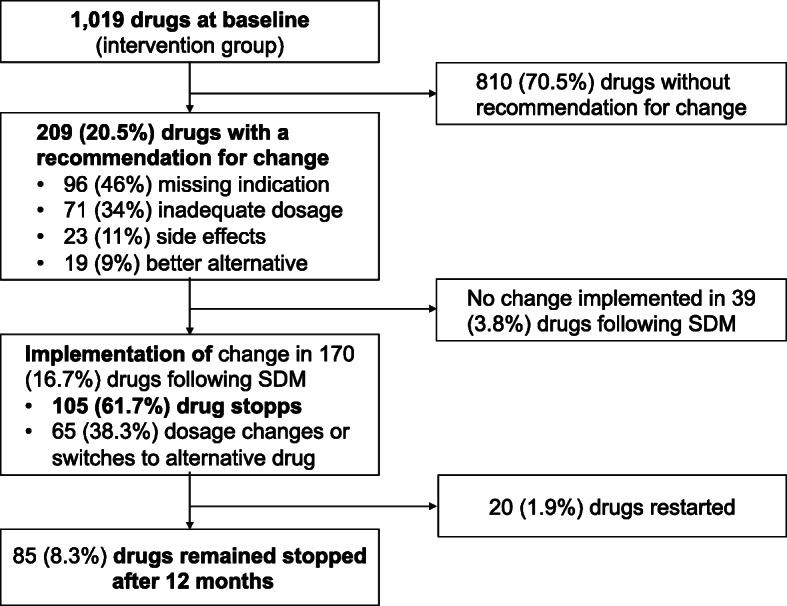
Drug flow chart. Drug charges in the intervention group after a recommendation by the PCP was given

PCPs reported that no drugs were taken without their previous knowledge at Pre-Intervention. For more information on drug classes that were stopped, restarted and started due to a new indication, see Fig. 5 and Additional file 5.

During the follow-up period, 43 (33.6%) of patients were hospitalised in the intervention group compared to 54 (26.2%) in the control group (*p* = 0.32 for between-group difference), and seven (5.5%) patients died in the intervention group compared to 13 (6.3%) in the control group (*p* = .95 for between-group difference) See Table 2 for details.

**Table 2:** Events and event rates

	Intervention group (*n* = 128)	Control group (*n* = 206)	p-value
**Clinical events** No. (%)	58 (45.3)	61 (29.6)	0.318
**Hospitalisations** No. (%)	43 (33.6)	54 (26.2)	0.162
**Deaths** No. (%)	7 (5.5)	13 (6.3)	0.954

The number of clinical events, hospitalisations, and deaths in both groups and significance between groups

Mean QoL values on 0–100 point scale at the three-time points were between 65.9 and 67.2 for the intervention group and between 65.0 and 69.0 for the control group, with no significant difference between groups (p from 0.270 to 0.383) and between time points. A significant increase in the QoL value in the control group between baseline and after 12 months (*p* = 0.025) was the only exception. Mean QoL on Likert-scale, and functional EQ-5D-3L showed no significant difference between time points or groups. For further details, see Table 3.

**Table 3:** Quality of life

	First consultation (T0)	6 months (T2)	6 months (T3)
**QoL (Likert-scale)**			
Intervention group	0.59 (0.95)	0.56 (0.99)	0.58 (0.93)
Control group	0.59 (1.00)	0.64 (0.95)	0.66 (1.03)
**QoL (VAS)**			
Intervention group	67.1 (18.11)	65.9 (17.84)	67.2 (17.13)
Control group	65.0 (17.71)	67.9 (16.99)	69.0 (17.39) *
**QoL (Functional EQ-5D-3L)**			
Intervention group	1.49 (0.36)	1.48 (0.36)	1.46 (0.35)
Control group	1.47 (0.34)	1.43 (0.31)	1.43 (0.33)

Comparison of QoL between groups and different consultations. Numbers given as mean and Standard Deviation (SD). * *p* < 0.05 (within group comparison between T0 and T3; control group). There is no significant difference between the two groups at any time point. Abbreviations: T0 = First consultation and baseline, T2 = consultation 6 months later, T3 = consultation 12 months later, QoL (Likert-Scale) = scale from − 2 - + 2 (− 2 = the worst possible – + 2 = the best possible) QoL (VAS) = scale from 0 to 100 (0 = the worst possible health status – 100 = the best possible health status) and QoL (Functional EQ-5D-3L) = scale from 0 to 3 (0 = no restriction – 3 = maximum restriction)

Overall time consumption of PCPs and nurses during the first consultation was 30.1 (SD 10.7) minutes in the intervention and 27.1 (SD 13.84) minutes in the control group (*p* = 0.066).

## Discussion

In our study population of older community-dwelling patients with polypharmacy, the use of a straight-forward and patient-centred deprescribing procedure was effective immediately after the intervention, but not after 6 and 12 months) compared to the control group providing usual care. Clinical events, hospitalisations and death rates, as well as QoL measures, were not significantly different between groups at all time points.

### Effectiveness over time

Our intervention resulted in a statistically significant immediate reduction in the number of drugs per patient, and 81% of all drugs stopped during the intervention consultation remained stopped after 12 months. However, the reduction in the total number of drugs was not preserved over time due to new prescriptions.

A probable reason might be our study population of old and multimorbid patients. As previously shown by Lam and others, this population is at high risk for new diseases, clinical events, hospitalisations and symptoms. Almost two-thirds of all patients faced either a clinical event or hospitalisation during the follow-up period, leading to new drugs and multiple drugs changes [51–53] (Table 2, Additional file 3). This natural course of diseases and consecutive drug treatment may dilute the overall impact of the intervention over time. As previously recommended by Dills et al., our results encourage *repeated* deprescribing interventions to reach a sustainable mid- and long-term effect, [54].

#### Safety and quality of life

Whenever deprescribing interventions are carried out, it is of utmost importance not to harm patients or deteriorate their QoL. As a non-inferiority safety measure, we found no negative impact of the intervention on clinical events, hospitalisations or deaths. Regarding QoL, resulting levels are in line with previously reported results in multimorbid patients in the Swiss primary care setting [55], but lower than those in the general population [32, 56–58]. Most importantly, the QoL results of the two groups in our study did not differ statistically significantly (Table 3)., thus reflecting that the deprescribing intervention was not inferior to usual care regarding QoL, i.e. did not lower the QoL of patients. Therefore we can conclude that the PCPs have deprescribed appropriately, without taking the risk of clinical or subjective deterioration of the patient.

However, the functional EQ-5D-3L QoL score decreased in both groups, likely due to the natural loss of function caused by ageing. (Table 3). As stated by Rickert et al. in their latest paper, the fact, the reduction of drugs did not increase harm to patients, can be seen as a positive result in itself [59].

#### Importance of SDM

The results of our study emphasise that an individualised approach and interaction with the patient is crucial for a successful and sustainable deprescribing. We ensured individualisation and interaction with the patient, by mean of addressing and prioritising patients’ subjective complaints in addition to medical diagnosis. Furthermore, we integrated SDM into the deprescribing algorithm. Our finding that patients agreed to a drug change in 86% of drugs recommended to change (on PCP level) indicates a high adherence towards the intervention, and we believe that this mainly due to focus on SDM during the deprescribing process. PCPs not only have frequent patient consultations and thus windows of opportunity to tackle polypharmacy together with their patient, but are also highly trusted by patients, as previously reported in a paper by our study group [60]. Mutual trust facilitates SDM procedures, resulting in more successful deprescribing [29, 60–63]. Von Buedingen et al., also using an SDM approach, reported a similar high adherence of patients towards their initial decision [52, 64, 65]. Page et al. came to a similar conclusion in their extensive systematic review on the feasibility and effect of deprescribing in older patients, highlighting the importance of patient-specific individualised approaches such as ours using SDM as the central mean of an intervention [18].

#### Barriers towards deprescribing

However, several psychological barriers are hindering a minority of patients from implementing drug changes [27, 66, 67]. We reported several reasons for a disagreement of or our study patients with the recommendations of PCP elsewhere [60]. The open discussion of deprescribing barriers is essential, to find solutions and to increase the efficacy of the deprescribing interventions [27]. This discussion and finding consent again is an integral part of the SDM process.

Time constraint is another major deprescribing barrier [17, 27, 35]. Our consultation duration was twice as long as an average encounter in the Swiss healthcare system, probably reflecting the task of dealing with multimorbid and complex patients, requiring more consultation time compared to the average ambulatory care population [68–70]. However, the duration did not differ significantly between groups. We conclude that our straight-forward study intervention itself did not require additional time which is supporting the feasibility of the intervention.

Another barrier is the fact that some PCPs may be reluctant to use a paper-based or digital “stand-alone” deprescribing algorithm. The integration of our procedure in existing electronic medical records and decision aids remains a promising option for further implementation while keeping in mind that this step is challenging and might induce potential new barriers [39, 59]_._

### Strengths

First, to our knowledge, the 12 months follow-up of this study is among the longest in deprescribing research and this setting. Second, we hardly selected our study population, resulting in a population of patients mainly living independently at home. Thus, thinking in terms of dissemination and public health impact, our findings may help to deal with older outpatient PCP population with polypharmacy.

### Limitations

First, we cannot rule out a selection bias of PCPs with a commitment for deprescribing and patient-centred communication style above-average volunteering more frequently for study participation. Otherwise, we believe the trial findings are generalisable for older patients in an industrialised outpatient primary care setting. Thus one must always bear in mind that the primary care setting is quite different between countries. Second, although the cluster-randomisation performed on PCP level resulted in an equal number of PCPs in both groups, the number of patients in the two groups varied noticeably, with fewer patients in the intervention group. Besides the fact that the protocol defined a maximum number of patients per PCP but not a minimal one, there is no apparent reason for this. However, we cannot rule out a potential association between the intervention procedure and the smaller number of patients in the intervention group. If we assume causality, this would be a limitation regarding the feasibility of the intervention. Third, we did not control for continuous patient recruitment by PCPs, thus cannot rule out a selection bias on patient level. Fourth, we cannot exclude an underreporting bias of clinical events, as long-term impacts of discontinuing drugs may sometimes require a longer time than our follow-up of 12 months.

## Conclusion

Our straight-forward and patient-centred deprescribing procedure is effective immediately after the intervention, but not after 6 and 12 months. Finding the ideal interval for repetition of deprescribing interventions will allow archiving sustainable mid and long term effects. Integrating SDM in the deprescribing process is a crucial factor for success.

## Supplementary Information

**Additional file 1.** PCP consultation checklist. Checklist PCPs of the intervention group used as a guideline and protocol for the first consultation. They documented all pre-interventional medication as well as PCPs recommendation, the PCPs reason for the recommendation and the shared-decision taken by the PCP and the patient.

**Additional file 2.** Baseline characteristics. Patient and PCP characteristics at baseline, thereby comparing patients who dropped out to those who finished the study. a) Likert scale ranging from − 2 to + 2, (− 2 the worst possible, + 2, the best possible). b) Visual analogue scale (VAS) ranging from 0 to 100, (0 the worst possible, 100 the best possible) c) EQ-5D-3L scale from 1 to 5 (1 for no problems to 5 extreme or being unable to perform a task).

**Additional file 3.** Drug charges in the intervention group. All drugs of invention group patients at baseline and changes due to the invention and during the follow-up.

**Additional file 4.** Deprescribing rates over time. Deprescribing rates over time between the two groups at different time points. The deprescribing rate was defined as the proportion of the number of drugs stopped at a given time point in relation to the number of drugs at baseline*.* N.a. = Not available.

**Additional file 5.** Overall drug changes during the study. All options a drug could take during the follow-up time of 12 months. Rows 5 to 8: pathways where drugs were stopped due to the actual intervention, + = drugs listed at this time point, − = drugs not listed at this time point, n.a. = not applicable. Example how to read the table: Option 5 (+/−/+/+) shows the pathway and the number of drugs stopped due to the intervention but restarted after 6 months, and still in place after 12 months.

**Additional file 6.** Anonymised data set. A minimal anonymised data set, necessary to replicate our study findings. An additional data description is included.

## Data Availability

All data generated or analysed during this study are included in this published article and its supplementary information files. We will additionally share a minimal anonymised data set necessary to replicate our study findings. See Additional file 6, which includes an additional data description.

## Abbreviations

DPP: drugs per patient
GPGP: Good Palliative-Geriatric Practice
IQR: interquartile range
PCPs: primary care physicians
QoL: quality of life
SDM: Shared decision making
SD: standard deviation
VAS: visual analogue scale
