# Supplementary figures and images for: Effect of a patient-centred deprescribing procedure in older multimorbid patients in Swiss primary care - A cluster-randomised clinical trial
Source: BMC Geriatr. 2020 Nov 16;20:471. doi: 10.1186/s12877-020-01870-8 (PMC7670707; doi:10.1186/s12877-020-01870-8)

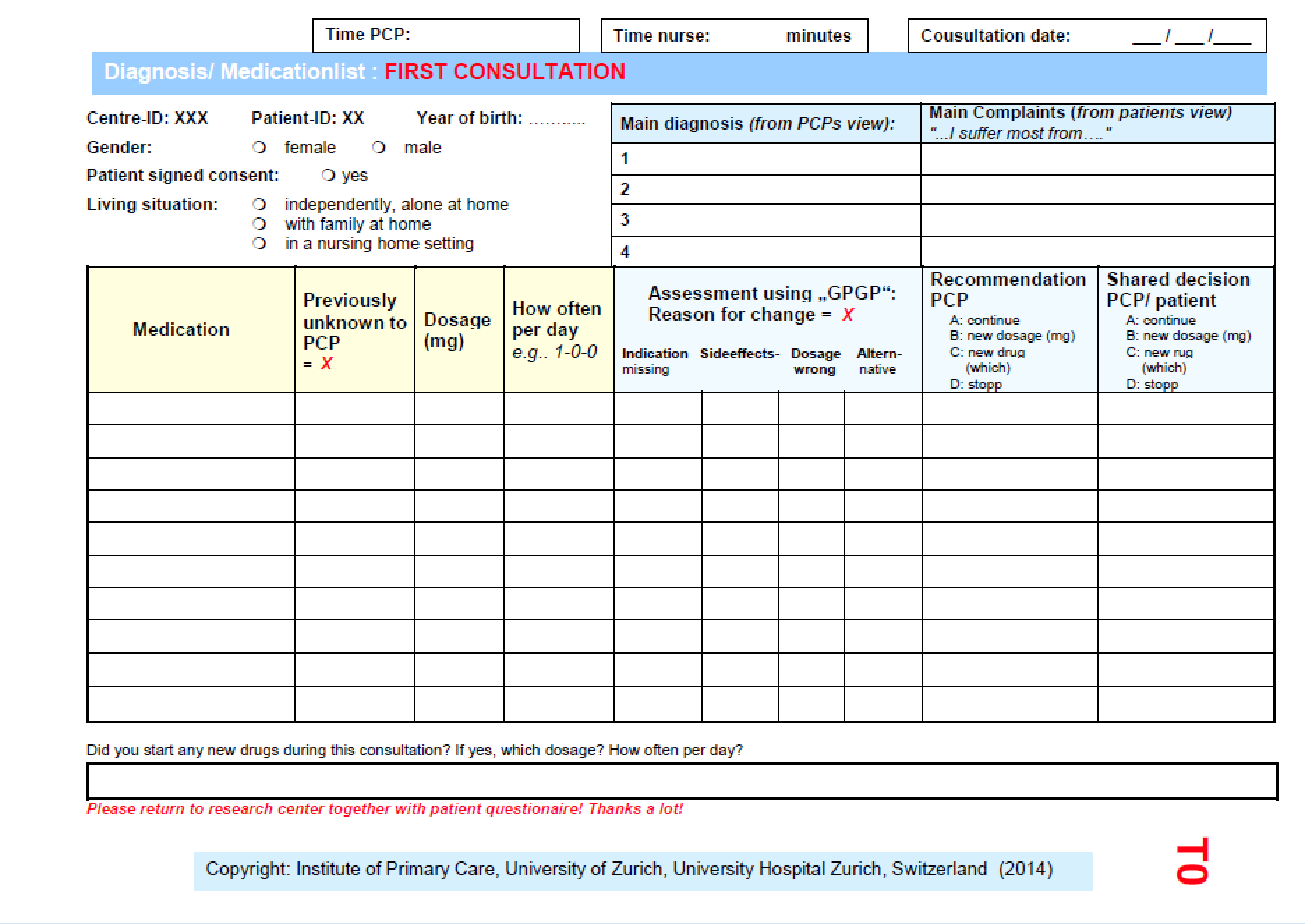

Supplement: Supplementary file 1 — Additional file 1. PCP consultation checklist. Checklist PCPs of the intervention group used as a guideline and protocol for the first consultation. They documented all pre-interventional medication as well as PCPs recommendation, the PCPs reason for the recommendation and the shared-decision taken by the PCP and the patient. [file 12877_2020_1870_MOESM1_ESM.png]
