# Supplementary material for: Effect of a patient-centred deprescribing procedure in older multimorbid patients in Swiss primary care - A cluster-randomised clinical trial
Source: BMC Geriatr. 2020 Nov 16;20:471. doi: 10.1186/s12877-020-01870-8 (PMC7670707; doi:10.1186/s12877-020-01870-8)
Supplement: Supplementary file 2 — Additional file 2. Baseline characteristics. Patient and PCP characteristics at baseline, thereby comparing patients who dropped out to those who finished the study. a) Likert scale ranging from − 2 to + 2, (− 2 the worst possible, + 2, the best possible). b) Visual analogue scale (VAS) ranging from 0 to 100, (0 the worst possible, 100 the best possible) c) EQ-5D-3L scale from 1 to 5 (1 for no problems to 5 extreme or being unable to perform a task). [file 12877_2020_1870_MOESM2_ESM.docx]

|  |  | **Finished** | **Dropout** | p |
| --- | --- | --- | --- | --- |
| **Patients** |  | 303 | 31 |  |
| Group (%) | Control | 190 ( 62.0) | 18 ( 58.1) | 0.81 |
|  | Intervention | 115 ( 38.0) | 13 ( 41.9) |  |
| Age (SD) |  | 76.92 (8.38) | 79.83 (8.94) | 0.072 |
| Sex (%) | Female | 136 ( 45.0) | 16 ( 51.6) | 0.609 |
|  | Male | 166 ( 55.0) | 15 ( 48.4) |  |
| Living situation (%) | independently at home | 107 ( 35.8) | 12 ( 38.7) | 0.003 |
|  | living with family | 163 ( 54.5) | 10 ( 32.3) |  |
|  | nursing home | 29 ( 9.7) | 9 ( 29.0) |  |
| Number of drugs (SD) |  | 7.75 (2.39) | 8.74 (2.93) | 0.032 |
| Health Status |  |  |  |  |
| QoL (SD) ^a^ |  | 0.61 (0.98) | 0.44 (1.04) | 0.348 |
| VAS (SD) ^b^ |  | 65.99 (18.08) | 64.26 (15.89) | 0.607 |
| EQ-5D-3L questionaire ^c^ |  |  |  |  |
| Mobility (SD) |  | 1.61 (0.67) | 1.65 (0.55) | 0.78 |
| Pain (SD) |  |  |  |  |
| Self-care (SD) |  | 1.18 (0.62) | 1.26 (0.51) | 0.525 |
| Activity (SD) |  |  |  |  |
| Anxiety (SD) |  |  |  |  |
| **PCPs** |  |  |  |  |
| Age (SD) |  | 51.28 (9.33) | 48.10 (8.72) | 0.07 |
| Sex (%) | Female | 52 ( 17.2) | 6 ( 19.4) | 0.954 |
|  | Male | 251 ( 82.8) | 25 ( 80.6) |  |
| Working experience (SD) |  | 14.86 (10.50) | 11.53 (9.47) | 0.096 |
| Consultation time (SD) |  | 21.99 (10.95) | 21.06 (6.67) | 0.643 |
