# Supplementary material for: Effect of a patient-centred deprescribing procedure in older multimorbid patients in Swiss primary care - A cluster-randomised clinical trial
Source: BMC Geriatr. 2020 Nov 16;20:471. doi: 10.1186/s12877-020-01870-8 (PMC7670707; doi:10.1186/s12877-020-01870-8)
Supplement: Supplementary file 3 — Additional file 3. Drug charges in the intervention group. All drugs of invention group patients at baseline and changes due to the invention and during the follow-up. [file 12877_2020_1870_MOESM3_ESM.docx]

|  | **Pre-Intervention** | **Post Intervention** | **Stopped due to Intervention** | | **Restarted** | |
| --- | --- | --- | --- | --- | --- | --- |
| **Cardiovascular** | 386 | 341 | **45** | Antihypertensive (40) | **4** | 4 |
|  |  |  |  | Lipid-lowering (5) |  | 0 |
| **Neurologic-analgetic** | 141 | 125 | **16** | Neuropleptics (10) | **3** | 1 |
|  |  |  |  | Analgetics (6) |  | 2 |
| **Anticoagulant/ antithrombotic** | 112 | 103 | **9** | Antithrombotisc (9) | **0** | |
| **Gastroenterological** | 93 | 80 | **13** | Proton-pump-inhibiting (7) | **4** | 1 |
|  |  |  |  | Anti-diarhetic (3) |  | 1 |
|  |  |  |  | Anti-obstipation (0) |  | 0 |
|  |  |  |  | Other (3) |  | 2 |
| **Endocrine** | 85 | 82 | **3** | Antidiabetics (3) | **0** | |
| **Immuno-suppressive- respiratory** | 34 | 28 | **6** | Corticosteroids (4) | **4** | 3 |
|  |  |  |  | Anti-obstructive inhalators (2) |  | 1 |
| **Other** | 168 | 155 | **13** | Minerals (5) | **5** | **0** |
|  |  |  |  | Gynecologicals/ Urologicals (2) |  | 2 |
|  |  |  |  | Anti-anemic (2) |  | **0** |
|  |  |  |  | Musculoscelettal (4) |  | 3 |
