# Supplementary material for: Effect of a patient-centred deprescribing procedure in older multimorbid patients in Swiss primary care - A cluster-randomised clinical trial
Source: BMC Geriatr. 2020 Nov 16;20:471. doi: 10.1186/s12877-020-01870-8 (PMC7670707; doi:10.1186/s12877-020-01870-8)
Supplement: Supplementary file 4 — Additional file 4. Deprescribing rates over time. Deprescribing rates over time between the two groups at different time points. The deprescribing rate was defined as the proportion of the number of drugs stopped at a given time point in relation to the number of drugs at baseline. N.a. = Not available. [file 12877_2020_1870_MOESM4_ESM.docx]

|  | **Intervention group**  **Deprescribing-rate**  %, (95% CI) | **Control group**  **Deprescribing-rate**  %, (95% CI | **Difference between groups**  **Deprescribing-rate**  %, (95% CI | **P-value** |
| --- | --- | --- | --- | --- |
| **Pre-Intervention** (T0) | n. a. | n. a. | n. a. | n. a. |
| **Post-Intervention** (T1) | 9.6% (7.6 – 11.5) |  |  |  |
| **6 months** (T2) | 23.9% (21.2 – 26.7) | 14.0% (12.2 – 15.8) | 9.9% (6.6 – 13.2) | <0.001 |
| **12 months** (T3) | 29.0% (26.0 – 31.9) | 21.5%° (19.3 – 23.6) | 7.5% (3.9 – 11.2) | <0.001 |
