# Supplementary material for: Effect of a patient-centred deprescribing procedure in older multimorbid patients in Swiss primary care - A cluster-randomised clinical trial
Source: BMC Geriatr. 2020 Nov 16;20:471. doi: 10.1186/s12877-020-01870-8 (PMC7670707; doi:10.1186/s12877-020-01870-8)
Supplement: Supplementary file 5 — Additional file 5. Overall drug changes during the study. All options a drug could take during the follow-up time of 12 months. Rows 5 to 8: pathways where drugs were stopped due to the actual intervention, + = drugs listed at this time point, − = drugs not listed at this time point, n.a. = not applicable. Example how to read the table: Option 5 (+/−/+/+) shows the pathway and the number of drugs stopped due to the intervention but restarted after 6 months, and still in place after 12 months. [file 12877_2020_1870_MOESM5_ESM.docx]

| **Options** | **Pre-**  **Inter vention** | **Post-Inter vention** | **6**  **months** | **12**  **months** | **Intervention group**  **Total number of drugs during the study**  (n=1298)  No. (%) | **Control group**  **Total number of drugs during the study**  (n=2011)  No. (%) |
| --- | --- | --- | --- | --- | --- | --- |
| **1** | + | + | + | + | 601 (46.3) | 1112 (55.3) |
| **2** | + | + | + | - | 103 (7.9) | 196 (9.7) |
| **3** | + | + | - | + | 28 (2.2) | 27 (1.3) |
| **4** | + | + | - | - | 182 (14.0)) | 279 (13.9) |
| **5** | + | - | + | + | 12 (0.9) | n.a. |
| **6** | + | - | - | + | 8 (0.6) | n.a. |
| **7** | + | - | + | - | 8 (0.6) | n.a. |
| **8** | + | - | - | - | 77 (5.9) | n.a. |
| **9** | - | + | + | + | 0 | 0 |
| **10** | - | + | + | - | 0 | 0 |
| **11** | - | + | - | + | 0 | 0 |
| **12** | - | + | - | - | 0 | 0 |
| **13** | - | - | + | + | 105 (8.1) | 140 (6.9) |
| **14** | - | - | + | - | 56 (4.3) | 110 (5.5) |
| **15** | - | - | - | + | 118 (9.1) | 147 (7.3) |
